# Humanization of care in pediatric wards: differences between perceptions of users and staff according to department type

**DOI:** 10.1186/s13052-020-00824-5

**Published:** 2020-05-19

**Authors:** C. Mandato, M. A. Siano, A. G. E. De Anseris, M. Tripodi, G. Massa, R. De Rosa, M. Buffoli, A. Lamanna, P. Siani, P. Vajro

**Affiliations:** 1 Pediatria Sistematica AORN “Santobono-Pausilipon”, Via Fiore, 6, Naples, Italy; 2 Cattedra di Pediatria - Dipartimento Medicina, Chirurgia e Odontoiatria “Scuola Medica Salernitana” Università di Salerno, UNISA, Baronissi, Salerno Italy; 3 Pediatria AOU “S. Giovanni di Dio e Ruggi D’Aragona”, Salerno, Italy; 4 Clinica Pediatrica AOU “S. Giovanni di Dio e Ruggi D’Aragona”, Salerno, Italy; 5 Dipartimento di Architettura, Ingegneria delle Costruzioni e Ambiente Costruito Politecnico di Milano, Milan, Italy; 6 Agenzia Nazionale per i Servizi Sanitari Regionali. AGENAS, Rome, Italy

**Keywords:** Humanization of care, Pediatric wards, Facilities, Perception, Users and staff

## Abstract

**Background:**

As the quality and quantity of patient-centered care may be perceived differently by recipients and independent observers, assessment of humanization of pediatric care remains an elusive issue. Herein we aim to analyze differences between the degrees of verified *existing* vs. *perceived* humanization issues of a pediatric ward. Furthermore, we examine whether there is concurrence between the degrees of humanization *perceived* by users (parents/visitors) vs. staff members.

**Methods:**

The study was conducted in the pediatric wards of seven medical centers of the Campania region (Italy) categorized as general (*n* = 4), children’s (*n* = 1), and university (*n* = 2) hospitals. The degree of *existing* humanization was assessed by a multidisciplinary focus group for each hospital through a pediatric care-oriented checklist specifically developed to individuate the most critical areas (i.e., those with scores < 2.5). The degree of *perceived* humanization was assessed through four indicators: well-being, social aspects, safety and security, and health promotion.

**Results:**

The focus groups showed that critical areas common to all centers were mainly concerned with welfare, mediation, translation, and interpretation services. Specific critical issues were care and organizational processes oriented to the respect and specificity of the person and care of the relationship with the patient. *Perceived* humanization questionnaires revealed a lack of recreational facilities and mediation and translation services.

As for specific features investigated by both tools, it was found that mediation and interpretation services were lacking in all facilities while patient perceptions and observer ratings for space, comfort, and orientation concurred only in the general hospital evaluations.

**Conclusions:**

Future humanization interventions to ensure child- and family-friendly hospital care call for careful preliminary assessments, tailored to each pediatric ward category, which should consider possible differences between perceived and verified characteristics.

## Background

The concept of “humanization of care” (HOC) in medicine is still not clearly defined; it is a multidimensional construct made up of several interactive factors within a process that is constantly evolving in relation to the changing needs of the patient and context of care [1, 2]. In general, HOC identifies the patient as a person in his/her wholeness as the focal point of the medical team. In pediatrics, HOC measures strive to provide care focused not only on the child patient but also on his/her family, all members of which are considered recipients [3].

North American (USA), South American (Brazil), and European agencies have elaborated and developed broad HOC programs [4]. The North American and European models focus specifically on the pediatric age, and their patient centeredness emphasizes the importance of the child–family dyad, including their active participation in medical decisions during hospitalization (e.g., through family-centered rounds, FCR). In contrast, the Brazilian model is part of a larger national program aimed at all age groups. However, despite the diversity of pediatric care across the world, there is a common need for improvement in the quality of interventions offered [5]. In order to satisfy this need, each distinct facility tries to implement its own local HOC measures. Although there is an absence of robust trials, a recent analytical review found that these measures are generally considered effective and likely to have beneficial effects on several aspects of pediatric hospitalization [6]. However, the reliability of the assessment of the studies under examination remains a vague issue requiring further research. In fact, patient and professional reports of the quality and quantity of HOC/person-centered care components may differ from those of independent observers [7, 8].

To answer the question regarding whether and to what extent there is concurrence between *existing* and *perceived* degree of HOC in pediatric facilities evaluated by specific tools, the present study investigated assessment differences regarding HOC acquired from focus groups in seven pediatric wards [9], as perceived by staff vs. parents [10], and depending on the category of pediatric setting being evaluated.

## Methods

Between July 2017 and October 2018, we studied seven pediatric wards reflecting three different categories of regional medical centers: children’s hospital [*n* = 1 (A)], pediatric department of a university hospital [*n* = 2 (B and C)], and general hospital [*n* = 4 (D, E, F, G)] (Table S1).

The first group represents a pediatric setting characterized by a medium-high level of general pediatric assistance. The second group represents a more specialized setting in the context of a university department. The third represents a limited pediatric context.

**1.**To assess *the****degree of existing HOC***, a pediatric-oriented inventory was specifically developed in collaboration with the National Agency for regional health services (AGENAS) based on an existing validated national checklist [9]. It is structured into **four core areas** (1.Care and organizational processes oriented to the respect and specificity of the person; 2.Physical accessibility, livability, and comfort of the places of care; 3.Access to information, simplification, and transparency; and 4.Care of the relationship with the patient) and **12 sub-areas** further divided into **28 criteria** and **122 items** [9]. It was accurately filled in by a focus group (one for each hospital) comprising representatives of four categories (medical staff, nursing staff, health management, and voluntary associations). Each item could receive a score from 0 to 10. The arithmetic means obtained in each area and in each criterion were calculated. According to the AGENAS, average scores (< 2.5) were considered “critical” and in need of interventions to improve the degree of existing HOC [9].

***2.The rating of perceived HOC*** was evaluated through the Listening to People to Cure People (LpCp)-tool [10], which consists of three short questionnaires (available from the authors on request) addressed to patients, visitors, parents, companions, staff, and technical evaluators. The survey includes an introductory section to acquire general information of the interviewed person (gender, age, nationality, occupation role, etc.) followed by a section investigating four indicators of users’ perceptions and experiences in the hospital [a) Well-being (comfort of the environment, recreational activities, and sports); b) Social aspects; c) Safety and security; and d) Health promotion (for technical evaluators only)].

Each **indicator** was assessed through a group of related questions, the answers to which present four levels of satisfaction (very satisfactory, fair, not very satisfactory, or unsatisfactory). The answers very satisfactory/fair and not very satisfactory/unsatisfactory were considered as positive and negative answers, respectively. An Excel spreadsheet elaborates the answers given by assigning a score to each theme based on the amount of positive answers obtained out of the total number of valid answers, with the following limits: full score, half score, and no score when positive answers were > 66%, 33-66%, and < 33%, respectively. The sum of the scores obtained amounts to the indicator’s *final score* (from 0 to 5 points total).

The hospital facility’s *final evaluation score* (from 0 to 100 points) is calculated as the weighted amount of scores achieved in all four indicators. The process of calculation considers the user-given and health care facility’s incidence on the improvement, looking at a minimum resource cost. The weight of the different indicators used by the tool was evaluated as shown in Table S2.

Areas scoring > 50% negative answers were considered “critical”, that is, as having the need for possible improvements by increasing reception and comfort quality.

In order to be effective, the tool must be distributed to a large percentage of hospital personnel (at least 10% of medical personnel and three evaluating technicians of a facility) and 10% of the parents of patients, based on the average number of daily patients.

## Results

### Degree of existing HOC (AGENAS checklist)

The items that obtained the lowest scores in different areas in the seven departments are summarized in Table 1.

**Table 1:** Items obtaining the lowest scores (gray boxes) in the different areas in the seven (A to G) wards* according to the National Agency for Regional Health Services (Agenas) checklist

	Items	Departments’ lowest scores						
		A	B	C	D	E	F	G
*Area 1*	1.1.1 Psychological support function	6.0	0	6.0	5,6	5.0	5.0	5
	1.1.4 Hospital without pain	4,6	6,5	2,6	1,6	1,2	4,3	2,8
	1.2.2 Respect for privacy	2,2	2.0	0	0	2.0	3,3	3,3
	1.3.1 Respect for linguistic specificities	2.0	0	0	6.0	0	0	0
	1.4.1 Continuity of care	1,7	1,7	0,5	0	2,9	1,1	1,1
*Area 2*	2.2.1 Orientation and signage	0	0	6,67	10.0	0	0	0
	2.4.1 Comfort of waiting rooms	5,7	0	0	1,4	8,5	5,7	4,2
*Area 3*	3.2.2 Access to information	2,5	1	4,5	6,5	5,5	0,5	3,5
*Area 4*	4.1.1 Communication care	2,8	1,6	4,3	1,8	5,7	1,4	1,4
	4.2.3 Staff training	0	0	0	0	0	0	0

Overall, the most critical issues that emerged in the seven departments concern:

- Area 1 ***(“****Care and organizational processes oriented to the respect and specificity of the person****”)*** that obtained scores ranging between 2.5 and 4. In particular, the items on psychological support function, hospitalization without pain, continuity of care, and respect for privacy and linguistic specificities obtained the lowest scores.
- Area 2 (“*Physical accessibility, livability, and comfort of the places of care*”) identified that the level of comfort at waiting rooms and orientation and signage in hospital was deficient in all facilities.
- Item 3.2.2 (Access to information) of Area 3 *(“Access to information, simplification, and transparency”)* obtained scores ranging between 0.5 and 6.5.
- Area 4 *(“Care of the relationship with the patient”)* with scores ranging between 2.5 and 6.7: staff training and communication care were poorly implemented aspects.

**Fig. 1:**
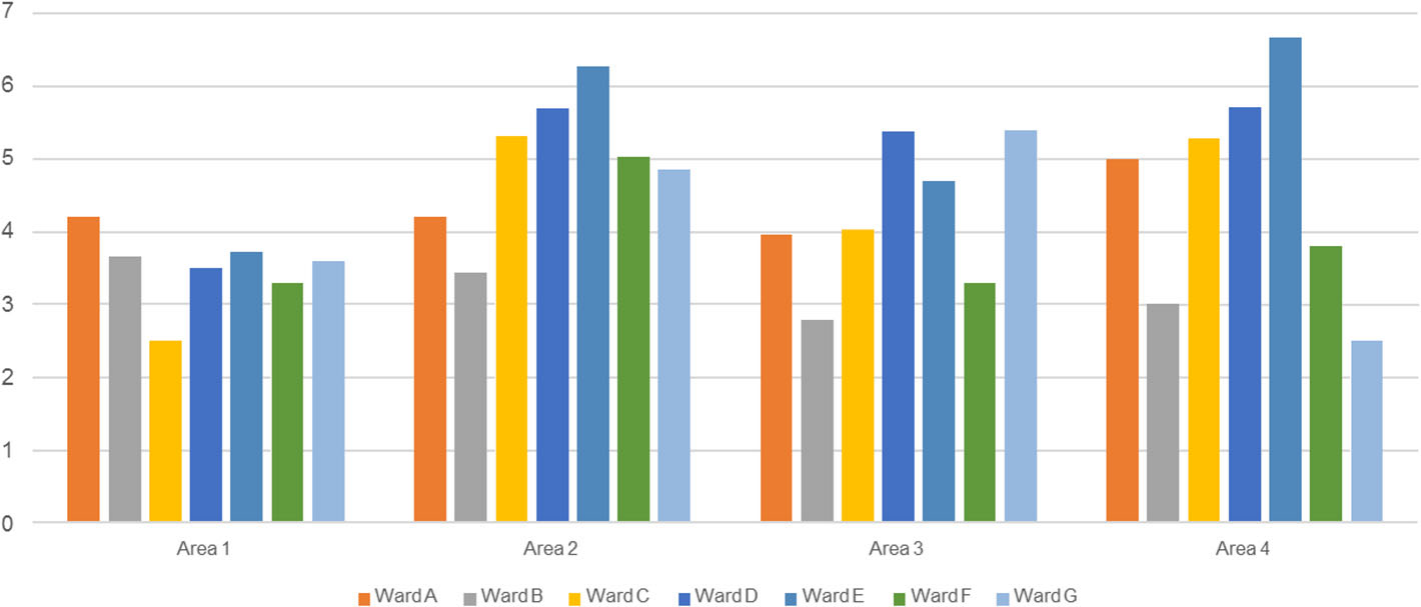
AGENAS pediatric checklist. Average values of scores obtained in each of the 4 areas of the *AGENAS* pediatric checklist for the seven pediatric wards analyzed. [Children’s Hospital (A), Pediatric Department of University Hospital (B and C) and General Hospital (D, E, F, G)]. The vertical axis indicate scores values 0–10. Area 1 (“Care and organizational processes oriented to respect and specificity of the person”). Area 2 (“Physical accessibility, livability and comfort of the places of care”). Area 3 (“Access to information, simplification and transparency”). Area 4 (“Care of the relationship with the patient”). Values < 2.5 are considered “critical”.

Average values obtained in each area of the AGENAS checklist for the seven wards analyzed are shown in Fig. 1**.** Altogether, the specific critical issues were regarding respect for anonymity, respect for linguistic specificities, continuity of care (including dialogue with the family pediatrician), and staff training. In addition, the equipment and characteristics of the hospital wards were not sufficiently “child-friendly,” although they were not included in the most critical items. (Data not shown; available on request.)

### Degree of perceived HOC (LpCp-tool)

**Fig. 2:**
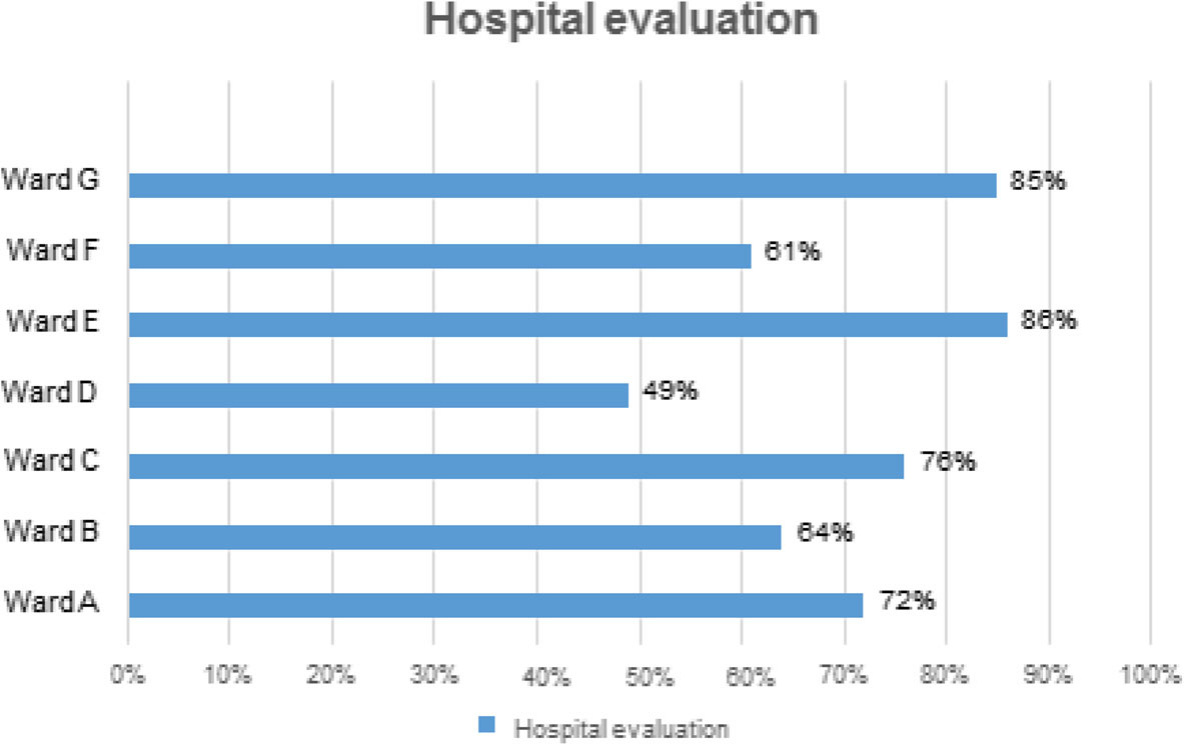
LpCp tool final score. Overall assessment of the *degree of humanization perceived by users and staff* of the seven pediatric wards examined with the LpCp tool. [Children’s Hospital (A), Pediatric Department of University Hospital (B and C) and General Hospital (D, E, F, G)]. The hospital facility’s final evaluation score (from 0 to 100 points) is instead calculated as the weighted amount of scores achieved in all four indicators. The process of calculation considers the user given and healthcare facility’s incidence on the improvement, looking at a minimum resource cost. The weight of the different indicators used by the tool are evaluated as shown in Table S2.

The analysis of the LpCp-tool results revealed the following information:

- *Well-being* was perceived by parents as critical in most of the seven facilities, although with some differences. In Ward D (general hospital), *parents/caregivers* had a generally negative perception of all aspects; the comfort of the rooms being the most inadequate (66.7% of negative responses; Fig. 1, Supplementary). In the remaining six wards, parents’/caregivers’ perception of the various aspects of well-being was quite regularly more positive (> 50% of positive feedback). The only exceptions regarded single aspects in Ward C (university department), concerning the (unquestionably) deficient presence of adjacent green areas, and in Ward F (general hospital), regarding the organization of recreational activities (Fig. 1, Supplementary).
  HOC ***perception****by the****staff*** was quite homogeneous in the seven departments and was generally negative regarding the poor organization of sports and recreational activities. However, the reduced comfort of the environments received more than 50% of positive feedback by the staff of Ward A (children’s hospital) and Wards E, F, and G (general hospitals)]. The aspect most positively judged by the staff of all seven wards was the orientation within the facilities (Fig. 2, Supplementary).
- *Social Aspects* received the highest percentage of positive responses from the parents/caregivers of all seven wards under review. In particular, the absence of discriminatory behavior toward patients and colleagues was the aspect perceived more positively by parents/caregivers and staff [with the exception of Ward E (general hospital) staff, which totaled about 65% of negative feedback] (Figs. 1, 2, Supplementary). The presence of mediation, translation, and interpretation services evaluated by the questionnaire for hospital staff received the highest percentage of negative responses as shown in Fig. 2 (Supplementary).
- *Safety:* In general, the safety aspect of all facilities was perceived positively by parents (> 50% of positive responses), with the exception of the presence of surveillance and the risk of infections, which were negatively perceived in Department D (general hospital). Security and safety were negatively perceived by the medical and nursing staff of all seven departments.
- *Campaigns for Health Promotion*: Organization of prevention and health promotion campaigns (questions addressed to assessment technicians) were unsuccessful in all seven departments under examination.

The final score obtained by each of the individual facilities is shown in Fig. 2. It was based on the weighted average of each criterion and indicated that the overall perception of the degree of HOC in the different departments in question was positive.

Regarding specific aspects investigated by both tools, in most cases**,** the ***existing*degree** of HOC did not concur with what was ***perceived***; that is, the lack of some resources was not evaluated negatively by parents and staff as one would expect (Table S3). While mediation and interpretation services evenly emerged as lacking in all facilities without inconsistencies between evaluators, parental perceptions and observer ratings *of* space, comfort, and orientation agreed in the general hospital evaluations but not in the two other settings (Table S4).

## Discussion

HOC during pediatric hospitalization is an important and, thus far, inadequately addressed issue. Individual aspects of HOC, namely family-centered care (FCC) and FCR in pediatric [11, 12] and/or in neonatal age and in a few specific subspecialty pediatric settings are available [13], but the overall understanding of the subject is still poor [14]. The problem is also complicated by the different possible perceptions/points of view on the measures adopted [7, 8]. Data from 469 healthcare providers were used to investigate the extent to which FCC principles are currently applied in clinical practice by healthcare providers working in inpatient units. Results showed that scores for daily FCC practices (current activities) were significantly lower than FCC practices performed for their perceived necessity (necessary activities) (*p* < .001) [15].

Recently, we tried to systematically review the value of a large spectrum of local interventions dealing with different aspects of HOC in general pediatric hospital wards. The results showed that HOC was considered central to the holistic management of pediatric hospital care; that most of the existing initiatives implemented in individual institutions/hospitals were not based on specific HOC models/programs; and that further and more robust research was needed for assessing their real importance [6]. Measuring the degree of HOC is crucial for setting priorities and intervention strategies to improve the quality of pediatric care. Currently available literature data summarized for pediatric aspects by Tripodi et al. [5] show that measurement tools used hitherto have been heterogeneous [16–18].

In general, the existing tools committed to HOC evaluation in various care settings (outpatient, day hospital, inpatient/hospitalization etc.) should relate to both the *objective evaluation* of the *existing* services offered, and the *perception of their quality* by a portion of users and healthcare workers, which have been rarely compared.

The main tools available to measure the different aspects of HOC [8, 16–18], unfortunately, are poorly comparable. For the assessment of the existing degree of hospital HOC, we used the pediatric version of a comprehensive checklist created by AGENAS specifically for Italian structures [9], which has been successfully used by other independent investigators in recent times to measure the degree of patient-centered care in a number of related structures before planning necessary improvement measures [19].

In association with the AGENAS checklist, we used the *LpCp-tool* [10], which was developed for the evaluation of the degree of perceived HOC as it is easy to understand and to fill in, as well as capable of involving different figures dealing with childcare in the hospital setting. As our study is the first time the LpCp-tool has been used in the pediatric field, the patient questionnaire had to be administered to patients’ parents.

Importantly, both tools used were applicable to different categories of pediatric facilities for identifying critical and implementable areas and allowed us to apperceive several facets of the same goal.

**Fig. 3:**
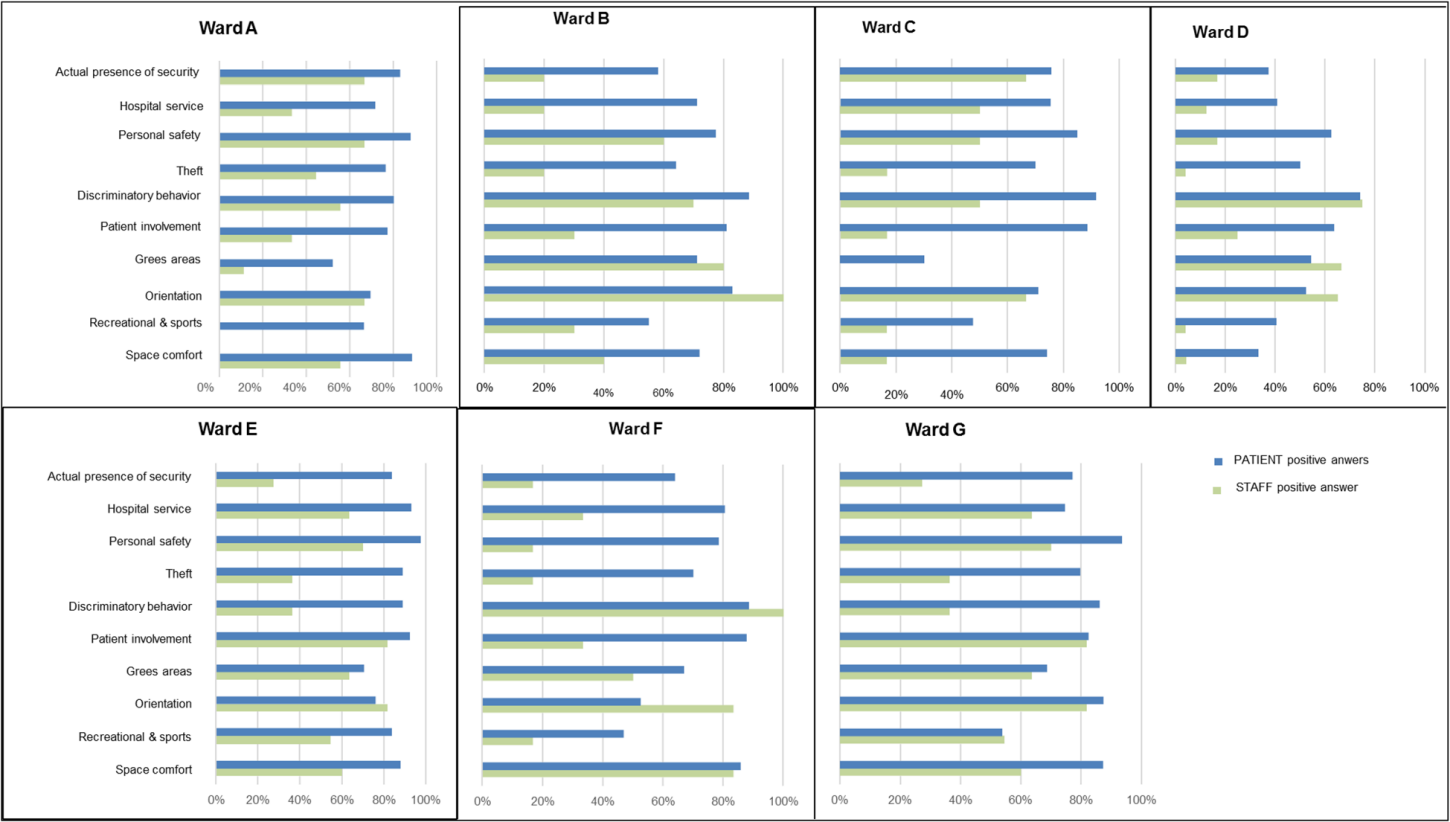
LpCp tool. Comparison of the percentages of positive responses given for each question by parents/companions (blue) and members of the staff (green) interviewed in each of the seven pediatric wards with LpCp tool. [Children’s Hospital (A), Pediatric Department of University Hospital (B and C) and General Hospital (D, E, F, G)]

The most critical issues that emerged from the analysis of our findings were related to the area of *wellbeing, safety, patient involvement in the therapeutic process, and physician involvement in the design proces*s. Interestingly, scarce agreement was found between the overall degree of HOC perceived by the staff and that perceived by parents in the facilities considered (Fig. 3). This confirms the trend observed in adult hospital settings in studies conducted with the same tool [7, 8, 10]. We believe that such a finding probably reflects healthcare staff’s superior knowledge of the real potential of the hospital vs. the opinion of users, who might tend to globally provide more positive responses on the basis of the healthcare received. Even the simple therapeutic communication and relationships between parents and nurses may improve the perception of the quality of care provided to children and their families [20]. Similarly, in another study, hospital employees scored hospital quality consistently lower than patients, and were also more heterogeneous in their assessments. Hospital size had no clear effect on the perception gap. Compared to patients and other employee groups, doctors have substantially different perceptions on hospital quality [21].

Finally, the results from the seven pediatric wards analyzed in our study seem to reflect the different categories of facilities. Children’s hospitals and the pediatric departments of university hospitals appear to have, by their nature, greater sensitivity and attention to the problems of the more frequently medium or long-term hospitalized child and of his/her family, which could justify the most positive perception of the users. However, two smaller general hospitals totaled the highest total score relative to the LpCp-tool. This could probably be explained by the recent structural improvements and a more serene climate due to the smaller size of the work department.

In sum, it is possible that the positive perception of the degree of HOC of the different facilities is influenced by the positive view of the users. Some aspects investigated by both tools (the AGENAS checklist and the LpCp-tool) could possibly hazard the comparison between the degree of *existing* and *perceived degree of* HOC. In most cases the *existing* did not concur with *what was perceived,* that is, the lack of some resources was not evaluated negatively as one would expect. However, a few exceptions emerged. For instance, mediation and interpretation services emerged as lacking in all the facilities without inconsistencies in both tools.

In the children’s hospital, space comfort and orientation, which received modest appreciation on the checklist, were not perceived very negatively by parents and staff. In small pediatric wards of general hospitals, space comfort and orientation received higher scores on the checklist (6.2 and 2.5 on average, respectively). In addition, users’ and staff perception was always positive (> 50% of positive responses) (Table S4).

## Study limitations

Family-centered, patient-centered, and collaborative approaches are now well established within the vocabulary of child healthcare. Children are central to this, yet their role within the FCC approach is not clear [22–24]. As parent and child experiences may differ, a major limitation of our study is the lack of direct evaluation of HOC by child and adolescent patients, the latter being a special population with significantly different healthcare needs. HOC for them needs a particular focus on the necessity of preserving personal privacy and autonomy with respect for their identity and to not adversely influence their recovery and dignity in general [25].

We are currently addressing this aspect by using the only available children’s tool developed in 2012 by the Health Promotion for Children and Adolescents by Hospitals Task Force for children aged 6–11 and 12–18 years [21], utilized so far only in a few Eastern European/Asian hospitals [24].

## Study strengths

This is the first study that attempted to evaluate the degree of existing and perceived HOC in the pediatric field. Identifying features that need to be improved in different pediatric care settings could be the first step in focusing attention on the HOC issue and implementing targeted interventions to create more child-friendly hospitals.

## Conclusion

Our study shows that the tools used are applicable to different categories of pediatric facilities for identifying target areas for improvement. The use of an evaluation tool with the achievement of measurable data is a sine qua non to allow any quantitative post-intervention verification of the effectiveness of the improvement actions undertaken. All in all, the greater perceived fulfillment of needs will probably be associated with greater participation in hospital care [26].

## Supplementary information

**Additional file 1 Figure S1 Supplementary.** LpCp tool for patients/visitors/parents/companions: percentages of positive responses (dark grey columns) given for each question by *parents/ companions* interviewed with the LpCp tool in each of the seven pediatric wards. [Children’s Hospital (A), Pediatric Department of University Hospital (B and C) and General Hospital (D, E, F, G)].

**Additional file 2 Figure S2 Supplementary.** LpCp tool for staff: percentages of positive responses (dark grey columns) given for each question by *members of the staff* interviewed with LpCp tool in each of the seven pediatric wards. [Children’s Hospital (A), Pediatric Department of University Hospital (B and C) and General Hospital (D, E, F, G)].

**Additional file 3 Table S1.** Pediatric wards enrolled, categorized in 3 different categories. **Table S2.** Weight of the different humanization indicators according to the Listening to people to Cure people (LpCp)-tool

**Additional file 4 Table S3.**Comparison between the degree of humanization existing and perceived, in the seven hospital wards enrolled.

**Additional file 5 Table S4.** Comparison between the degree of humanization existing and perceived, in the seven hospitals wards categorized under the three types of settings.

## Data Availability

Not applicable.

## Abbreviations

AGENAS: National Agency for Regional Health Services
FCC: Family Centered Care
FCR: Family Centered Rounds
HOC: Humanization of care
LpCp: Listening to People to Cure People
