# Supplementary figures and images for: Humanization of care in pediatric wards: differences between perceptions of users and staff according to department type
Source: Ital J Pediatr. 2020 May 19;46:65. doi: 10.1186/s13052-020-00824-5 (PMC7238599; doi:10.1186/s13052-020-00824-5)

Ward A

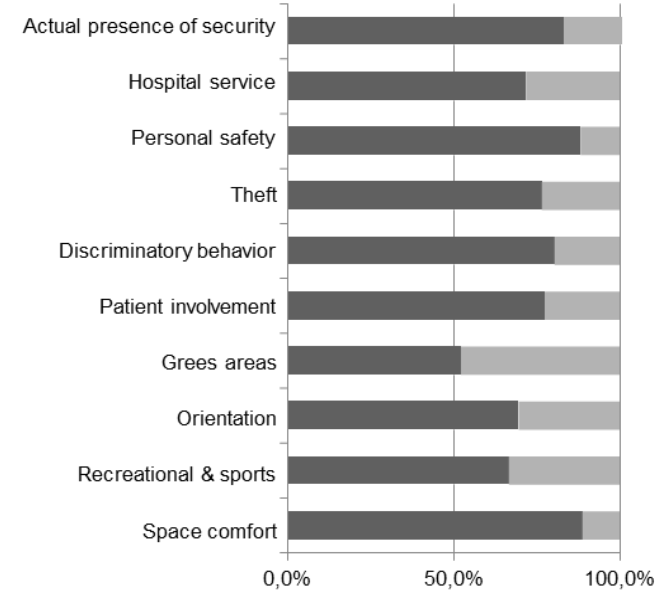

Ward B

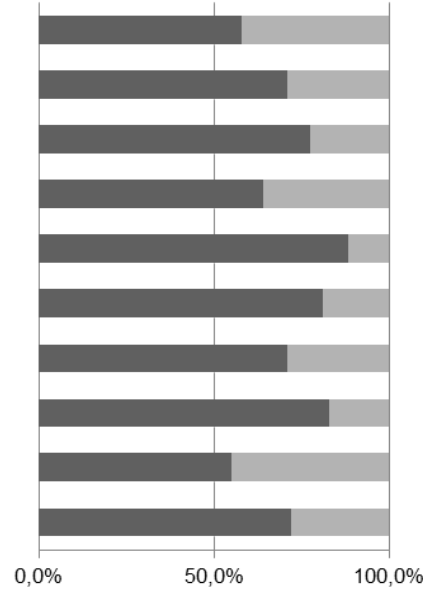

Ward C

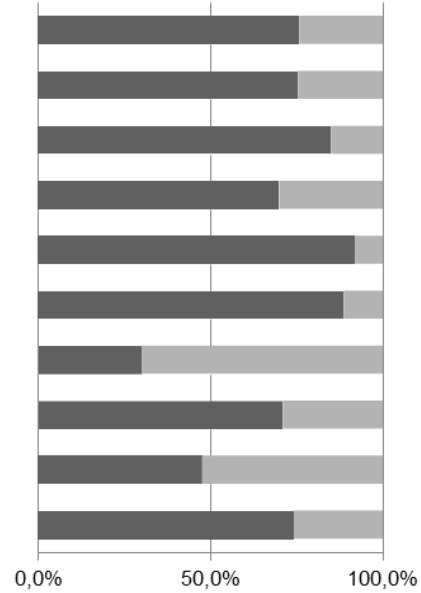

Ward D

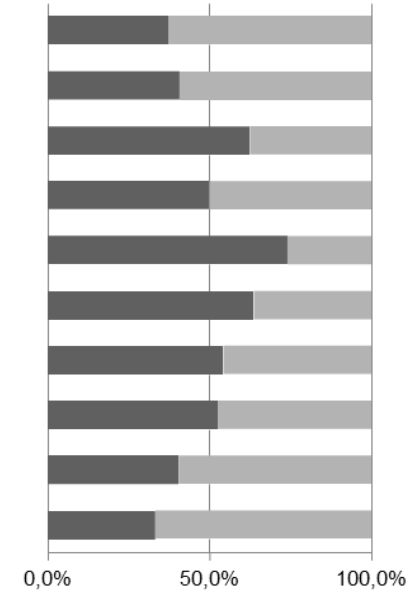

Ward E

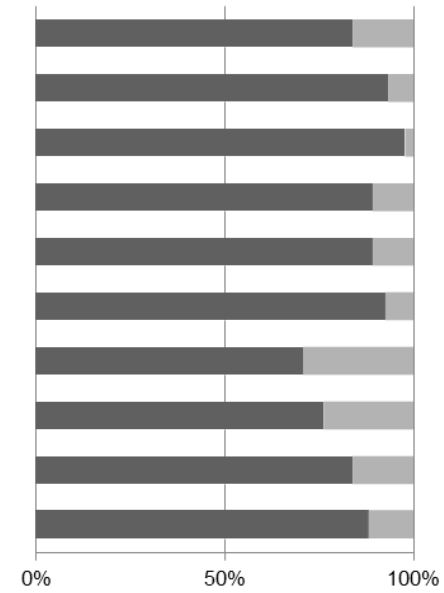

Ward F

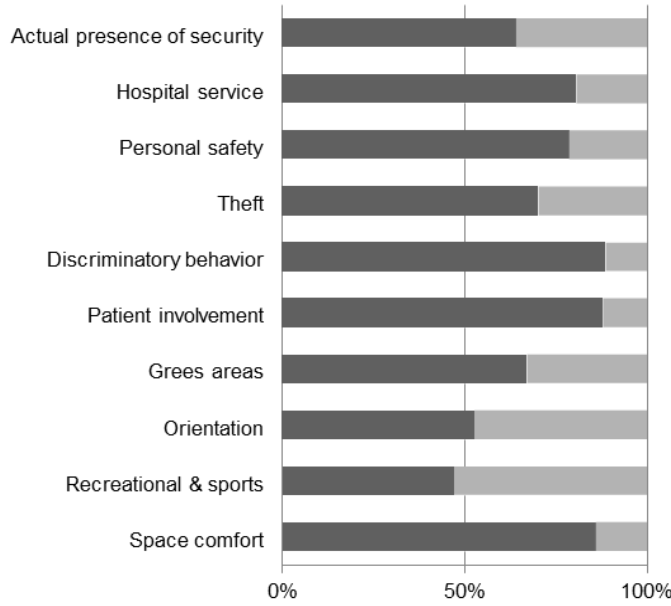

Ward G

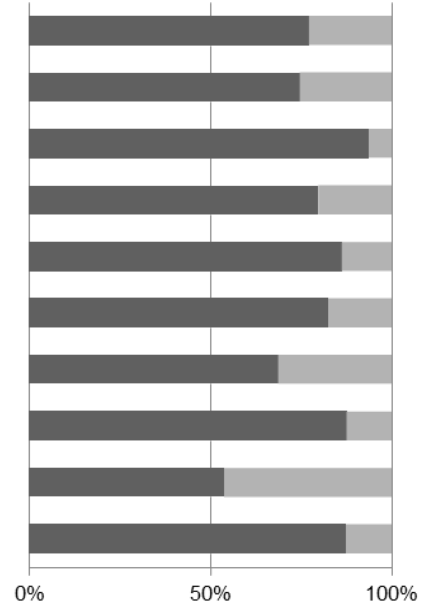

Figure 2

Supplement: Supplementary file 1 — Additional file 1 Figure S1 Supplementary. LpCp tool for patients/visitors/parents/companions: percentages of positive responses (dark grey columns) given for each question by parents/ companions interviewed with the LpCp tool in each of the seven pediatric wards. [Children’s Hospital (A), Pediatric Department of University Hospital (B and C) and General Hospital (D, E, F, G)]. [file 13052_2020_824_MOESM1_ESM.pdf]

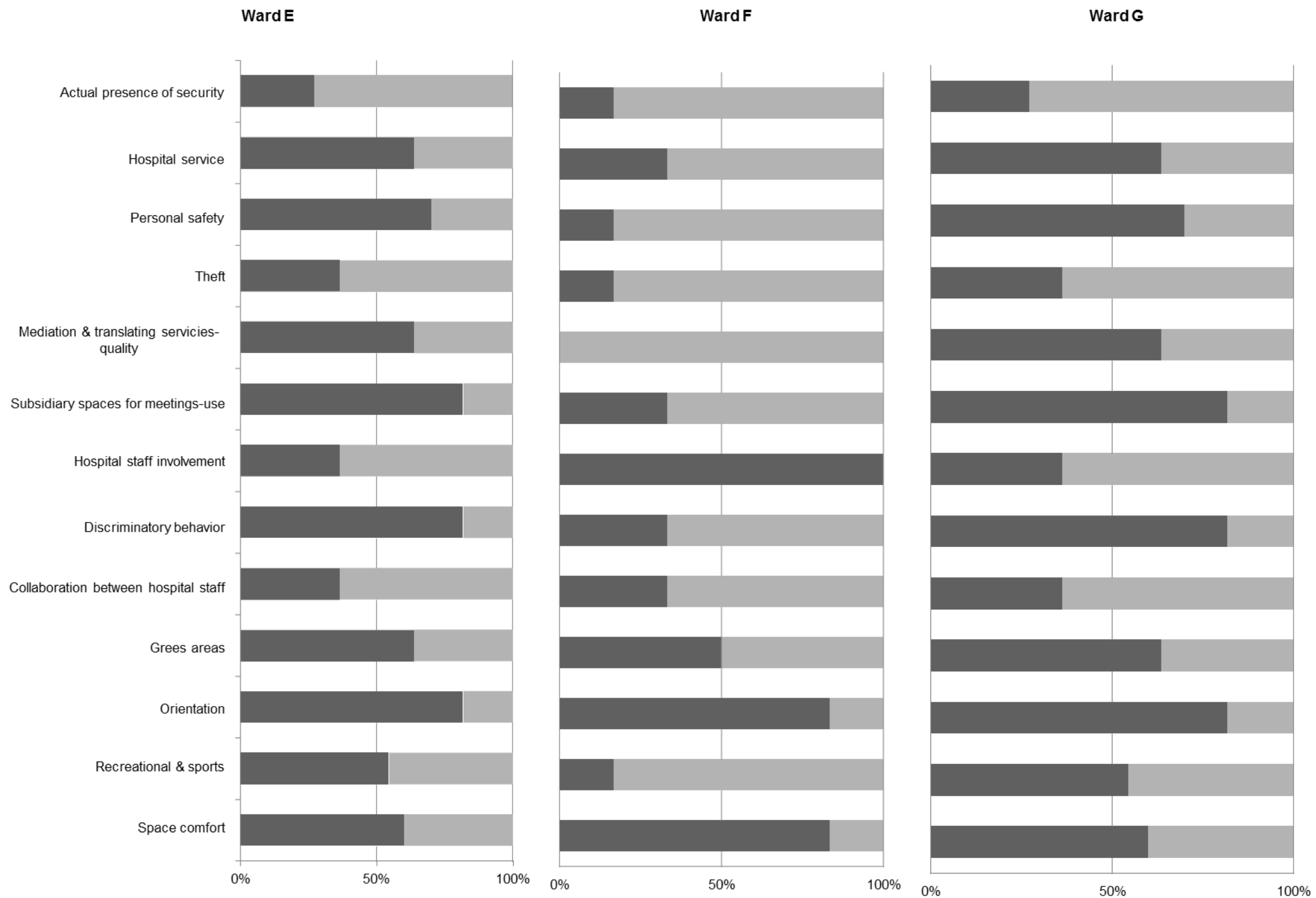

**Figure 3**

Supplement: Supplementary file 2 — Additional file 2 Figure S2 Supplementary. LpCp tool for staff: percentages of positive responses (dark grey columns) given for each question by members of the staff interviewed with LpCp tool in each of the seven pediatric wards. [Children’s Hospital (A), Pediatric Department of University Hospital (B and C) and General Hospital (D, E, F, G)]. [file 13052_2020_824_MOESM2_ESM.zip › MANDATO& SIANO FIGURE3b.pdf]

Ward A

Ward B

Ward C

Ward D

■ TOT positive answers ■ TOT negative

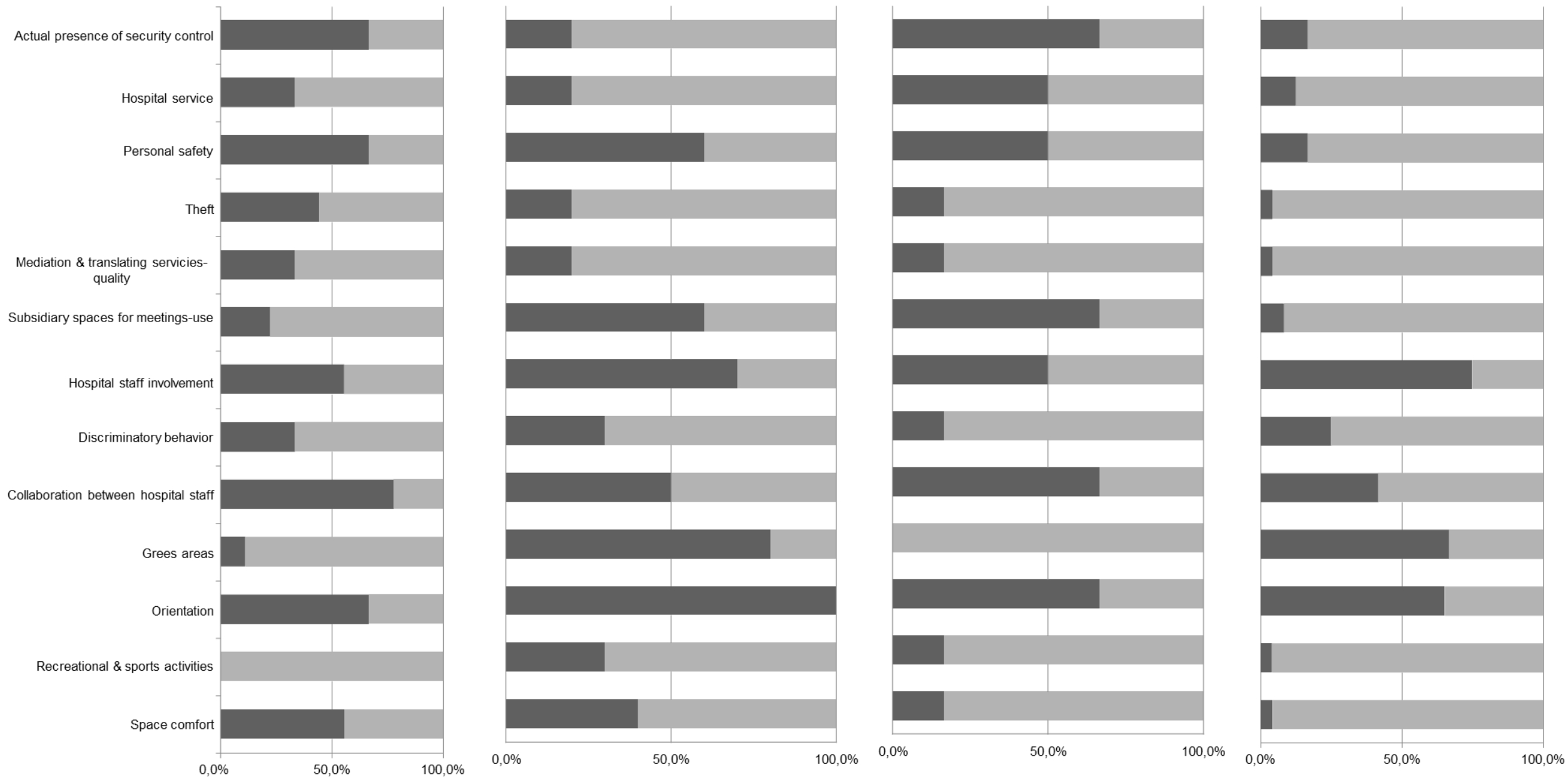

Supplement: Supplementary file 2 — Additional file 2 Figure S2 Supplementary. LpCp tool for staff: percentages of positive responses (dark grey columns) given for each question by members of the staff interviewed with LpCp tool in each of the seven pediatric wards. [Children’s Hospital (A), Pediatric Department of University Hospital (B and C) and General Hospital (D, E, F, G)]. [file 13052_2020_824_MOESM2_ESM.zip › MANDATO& SIANO FIGURE3a.pdf]
