# Supplementary material for: Humanization of care in pediatric wards: differences between perceptions of users and staff according to department type
Source: Ital J Pediatr. 2020 May 19;46:65. doi: 10.1186/s13052-020-00824-5 (PMC7238599; doi:10.1186/s13052-020-00824-5)
Supplement: Supplementary file 3 — Additional file 3 Table S1. Pediatric wards enrolled, categorized in 3 different categories. Table S2. Weight of the different humanization indicators according to the Listening to people to Cure people (LpCp)-tool [file 13052_2020_824_MOESM3_ESM.pdf]

Table S1. Pediatric wards enrolled, categorized in 3 different categories

Hospital	City	Facility category	N beds
General Pediatrics of AORN *“Santobono- Pausilipon”* **(A)**	Naples	Children Hospital	16 pediatric beds/287 total pediatric beds
Department of Pediatrics of AOU *“Federico II”* **(B)**	Naples	University Hospital	27 pediatric beds/ 47 total pediatric beds
Department of Pediatrics of AOU *“Luigi Vanvitelli”* **(C)**	Naples		12 pediatric beds/ 24 total pediatric beds
General Pediatrics of AOU *“S. Giovanni di Dio e Ruggi D’ Aragona”* **(D)**	Salerno	General Hospital	28 pediatric beds/ 642 total general beds
General Pediatrics of AO “*San Giuseppe Moscati”* **(E)**	Avellino		19 pediatric beds/ 483 total general beds
General Pediatrics of AO *“Sant’Anna e San Sebastiano”* **(F)**	Caserta		14 pediatric beds/ 486 total general beds
General Pediatrics of General Pediatrics of AO *“San Pio”* **(G)**	Benevento		16 pediatric beds/ 402 total general beds

Between July 2017 and October 2018, we studied seven pediatric wards reflecting three different categories of regional medical centers: children’s hospital [n=1 (A)], pediatric department of university hospital [n = 2 (B and C)], and general hospital [n= 4 (D, E, F, G)](Table S1).

Table S2 Weight of the different humanization indicators according to the Listening to people to Cure people (LpCp)-tool

INDICATOR	WEIGHT(%)
Well-Being	13
Social Aspects	38
Safety and Security	42
Health Promotion	7
**Total**	**100**
