# Supplementary material for: Humanization of care in pediatric wards: differences between perceptions of users and staff according to department type
Source: Ital J Pediatr. 2020 May 19;46:65. doi: 10.1186/s13052-020-00824-5 (PMC7238599; doi:10.1186/s13052-020-00824-5)
Supplement: Supplementary file 4 — Additional file 4 Table S3.Comparison between the degree of humanization existing and perceived, in the seven hospital wards enrolled. [file 13052_2020_824_MOESM4_ESM.pdf]

Table S3 Comparison between the degree of humanization existing and perceived, in the seven hospital wards enrolled

	Ward A			Ward B		
	Existing*	Perceived**		Existing*	Perceived**	
		Parents	Staff		Parents	Staff
Space Comfort	Criteria 2.3.1***=0,56<br>Criteria 2.3.2***=1,31	88,9%	55,6%	Criteria 2.3.1***=0,67<br>Criteria 2.3.2***=2,25	72%	40%
Orientation	Criteria 2.2.1***=0	69,7%	66,7%	Criteria 2.2.1***=0	83%	100%
mediation/translation /interpretation services	Criteria1.3.1***=2	/	33,3%	Criteria 1.3.1***=0	/	20%

	Ward C			Ward D		
	Existing*	Perceived**		Existing*	Perceived**	
		Parents	Staff		Parents	Staff
Space comfort	Criteria 2.3.1***=0,56<br>Criteria 2.3.2***=2,75	74,2%	16,7%	Criteria 2.3.1***=2,42<br>Criteria 2.3.2***=0,78	33,3%	4,3%
Orientation	Criteria 2.2.1***=6,67	71%	66,7%	Criteria 2.2.1***=10	52,5%	65,2%
mediation/translation /interpretation services	Criteria 1.3.1***=0	/	16,7%	Criteria 1.3.1***=6	/	4,2%

	Ward E			Ward F		
	Existing*	Perceived**		Existing*	Perceived**	
		Parents	Staff		Parents	Staff
Space Comfort	Criteria 2.3.1***=8<br>Criteria 2.3.2***=4.58	88%	60%	Criteria 2.3.1***=6.40<br>Criteria 2.3.2***=3.75	85,9%	83,3%
Orientation	Criteria 2.2.1***=0	76,1%	81%	Criteria 2.2.1***=0	52.5%	83,3%
mediation/translation /interpretation services	Criteria 1.3.1***=0	/	63,6%	Criteria 1.3.1***=0	/	0%

Ward G			
	Existing*	Perceived **	
		Parents	Staff
Space Comfort	Criteria 2.3.1***=8<br>Criteria 2.3.2***=3.33	87,3%	60%
Orientation	Criteria 2.2.1***=0	87,5%	81,8%
mediation/translation /interpretation services	Criteria 1.3.1***=0	/	63,6%

*average criteria scores; **% positive answers ***Criteria 1.3.1:respect for linguistic specificities; 2.2.1: orientation and signage; 2.3.1:equipment and characteristics of the wards; 2.3.2: specific features and characteristics of the wards

Gray boxes indicate critical criteria according to the National Agency for Regional Health Services (Agenas) checklist for the existing, or negative answers according to Listening to people to Cure people (LpCp)-tool for the perceived ..
